# The interest of ketamine as an adjuvant to fentanyl in post-tonsillectomy analgesia in children: a randomized controlled trial

**DOI:** 10.11604/pamj.2024.49.81.42791

**Published:** 2024-11-18

**Authors:** Imen Zouche, Salma Ketata, Ines Kharrat, Faiza Grati, Sirine Ayadi, Mariem Keskes, Rahma Derbel, Ilhem Charfeddine, Hichem Cheikhrouhou

**Affiliations:** 1 Department of Anesthesiology, Habib Bourguiba University Hospital, 3000, Sfax, Tunisia,; 2 Department of Oto-rhino-laryngology, Habib Bourguiba University Hospital, Sfax, Tunisia

**Keywords:** Tonsillectomy, children, postoperative pain, fentanyl, ketamine, analgesia

## Abstract

**Introduction:**

tonsillectomy is the most commonly performed surgery in children. It is a painful surgery, which is often an ordeal for both children and their parents. The study aimed to evaluate the effects of ketamine used instead of or as an adjuvant to fentanyl on early postoperative pain scores in children undergoing tonsillectomy.

**Methods:**

we conducted a double-blind prospective randomized study including 60 children, aged between 2 and 7 years, scheduled to undergo adenotonsillectomy. Patients were randomly assigned to one of three groups: group G1 received 2 μg/kg of fentanyl, group G2 received 0.5 mg/kg of ketamine and group G3 received an association of fentanyl 1μg/kg and 0.25 mg/kg of ketamine. We recorded postoperative analgesic requirements and side effects. The pain was assessed in the post-anesthesia care unit (PACU) by the face, legs, activity, cry, and controllability (FLACC) pain scale. We evaluated the requirement for additional analgesics, postoperative nausea, and vomiting.

**Results:**

sixty children were included. Twenty patients were randomly assigned to one of three groups. Better control of pain was noted in group G3 ( Median FLACC scale G3=0 Inter quartile range (IQR)=-1 - 1), with a significant difference at 30 min compared to both groups G1 (median FLACC scale G1=3 [IQR=-1 - 7]; p=0,008) and G2 (median FLACC scale G2=1 [IQR=-2 - 4]; p=0.036). The need for additional analgesia and side effects in the PACU were comparable for the three groups.

**Conclusion:**

ketamine associated with fentanyl provides satisfactory early analgesia and can even replace fentanyl during tonsillectomy.

## Introduction

Tonsillectomy continues to be the most commonly performed surgery in children. It is a painful surgery, which is often an ordeal for both children and their parents. Thus, controlling this suffering in children still poses therapeutic dilemmas [1]. Inadequate pain management after tonsillectomy may result in poor oral intake, dehydration, sleep disturbances, vomiting, and agitation in the first 24 hours after surgery [2]. Many therapeutic modalities as nonsteroidal anti-inflammatory agents, opioids, and local anesthetics have been used for pain management after tonsillectomy surgery in the literature. Given the simplicity of their administration, opioids are commonly used to manage most patients after tonsillectomy [3]. Nevertheless, morphine may cause nausea, vomiting, and significant respiratory problems, especially in children who have undergone tonsillectomy to treat obstructive sleep apnea [4]. Ketamine is a potent noncompetitive N methyl-D-aspartate (NMDA) antagonist that when added to general anesthesia at sub-anesthetic doses may reduce the postoperative analgesics consumption [5]. Compared with opioids, drowsiness, respiratory depression, and vomiting are less common with ketamine [6]. Our study aimed to evaluate the effects of ketamine alone or in combination with fentanyl on postoperative pain intensity and morphine consumption in a North African pediatric population undergoing tonsillectomy.

## Methods

We conducted a double-blind study including children proposed for elective tonsillectomy and adenoidectomy (adenotonsillectomy) under general anesthesia.

**Study population:** our study included children aged between 2 and 7 years, ASA I or II, and scheduled for elective adenotonsillectomy under general anesthesia. Non-inclusion criteria were children with allergies to the used drugs and those who had obstructive sleep apnea syndrome. We excluded patients who presented a surgical complication such as important bleeding and whose anesthetic or analgesic protocol was changed.

**Sample size:** a sample size of 18 patients in each group was calculated to detect a mean difference of 2 points in the FLACC scale at 30 min with α= 0.05 and β= 0.9 (the standard deviation from a pilot study was 1.8). Therefore, we decided to enroll 20 patients in each group to allow for possible dropouts.

**Randomization and allocation:** children were randomized using a computer-generated random number table into three groups each: group G1 received 2 μg/kg of fentanyl, group G2 received 0.5 mg/kg of ketamine and group G3 received an association of fentanyl 1μg/kg and 0.25 mg/kg of ketamine. To maintain the blindness of the study, an anesthesiologist, who was not involved in the anesthesia care prepared the solution based on the randomization list. During the entire study period, investigators performing the intraoperative and postoperative assessments, medical staff (nurse, anesthetist, and surgeon), subjects, and parents were not aware of the group allocation.

**Intervention:** all patients had a pre-anesthetic consultation and did not receive premedication. Anesthesia began with sevoflurane-oxygen mask anesthetic induction with 8% inspired sevoflurane before venous access followed by administration of the tested drug with an association of propofol (3mg/kg) and celocurin (1mg/kg) for intubation. All subjects had received one of the three interventions according to the randomization. The anesthesia maintenance was provided with sevoflurane at 3% and all the patients received a dose of paracetamol 15 mg/kg at the beginning of surgery. The surgical technique was standardized, using a standardized snare dissection technique. At the end of the surgery, anesthesia was discontinued and the tracheal tube was removed in the operating room when the patient demonstrated spontaneous eye opening and airway reflexes. All children were transferred to the post-anesthetic care unit (PACU) where standard monitoring was established, and they were observed for 2 hours.

**Data collection:** pre- and intraoperative data were collected including patients´ demographics, duration of surgery, and anesthesia. At the end of anesthesia, we noticed the time of eye-opening for each patient. Postoperative data were collected in the PACU: pain was assessed by the face, legs, activity, cry, controllability (FLACC) pain scale (which is a validated measure of pain for children in this age group) [7]. The FLACC includes five items scored 0-2 as identified in the name of the scale. We collected during the total stay in the PACU: the severity of pain (measured upon arrival in the PACU and every 30 minutes through the FLACC scale), the need for additional analgesia, and the occurrence of incidents during the stay in the PACU. As supplementary analgesia, a bolus of IV morphine 1 μg/kg, was given to patients with an FLACC scale >4. The anesthesiologists, PACU staff, and study personnel were blinded concerning group allocation. Nausea and vomiting were recorded on entry into the PACU and then at 30 min, 60 min, and 90 min. Arterial pressure, heart rate, oxygen saturation, and respiration frequency were also monitored every 30 min until discharge from PACU.

**Variables:** our primary outcome was the severity of pain assessed with (FLACC) score. The secondary outcome was: the time of eye opening for each patient. The incidence of postoperative vomiting, the request for additional analgesia, the preoperative and in PACU Arterial pressure, heart rate, oxygen saturation, and respiration frequency.

**Statistical analysis:** all variables including baseline characteristics were presented as a number with a percentage for categorical variables, mean ± standard deviation for continuous variables following a normal distribution, and median with interquartile range (IQR) for continuous variables not following a normal distribution. Normal distribution was tested with the Kolmogorov-Smirnov test. Statistical analysis was performed with SPSS 20 software for Windows. The data was analyzed using one-way ANOVA or Kruskal-Wallis test. When significant differences existed, the least-significant difference (LSD) was applied to detect the exact location among groups. The requirement for additional analgesics and the incidence of postoperative nausea and vomiting were compared using a Chi-square or two-tailed Fisher´s exact test. A p-value less than 0.05 was considered statistically significant.

**Ethical considerations:** the study was conducted after approval of the Southern Protection Committee of People (C.P.P.SUD) under the aegis of the Health Ministry of the Tunisian Republic reference CPP SUD N°18/14. Written, informed parental consent was obtained from all participants.

## Results

**Figure 1:**
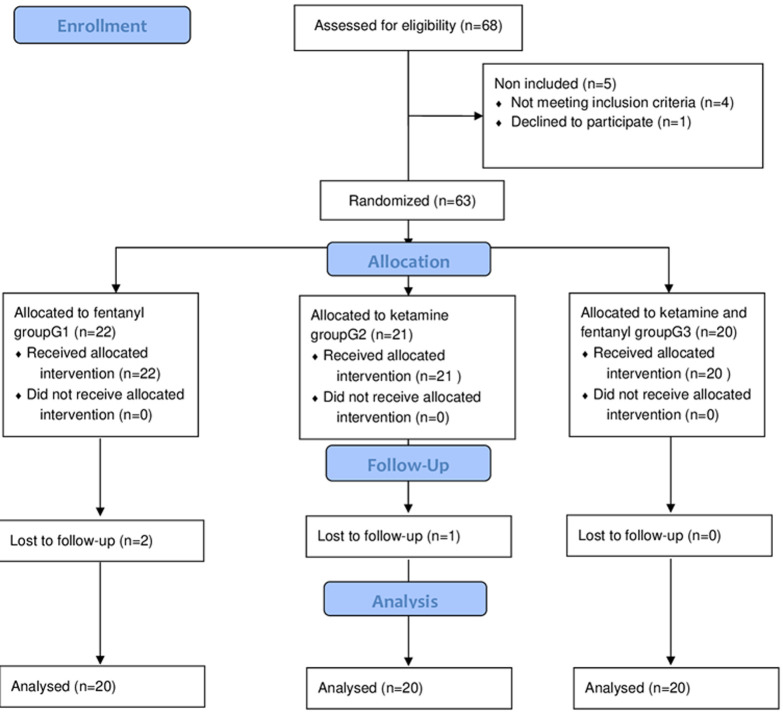
flow chart showing the eligible patients analyzed, and the randomization of the groups

We enrolled 63 children. Two children randomized to the group G1 and one child randomized to the group G2 were excluded because of missing data. Finally, 60 children were analyzed and divided into 3 groups: 20 children for each group (Figure 1). Sixty children were analyzed and divided into 3 groups of 20. No patients were excluded (Figure 1).

**General characteristics:** our sample was characterized by a median of age =5 years with extreme (3-7), a sex ratio (male/female) =1.22, a median of weight = 25 kg with extreme (12-30), and a height= 110cm with extreme (80-154). The demographic data of the children were not significantly different between the three groups (Table 1). The median age of the children was 4 years, 5 years, and 5 years for the groups G1, G2, and G3 respectively. Overall, the number of male patients was higher but with no statistically significant sex predominance among the three groups. The anesthesia and surgical time were found to be comparable among groups (Table 1). The average length of the surgery and anesthesia was 17 ± 5.6 min and 23 ± 6.2 min respectively. Eye-opening after anesthesia in minutes (16.1 ± 7.1 min for G1, 17.9 ± 5.6 min for G2, 20.6 ± 8.7 min for G3) was comparable among groups. The intraoperative hemodynamic parameters were comparable among the three groups.

**Table 1:** patient demographics, duration of anesthesia and surgery

	G1	G2	G3	P-value
Age (years)	4 (1)	5(2)	5 (2)	0.06
Sex (male: female)	10:10	9:11	14:6	0.2
Weight (Kg)	22.5 (7)	23.5 (10)	20 (9)	0.07
Length (cm)	105 (20)	120 (15)	120 (24)	0.6
Duration of surgery (minutes)	17.5 ± 4.7	17.9 ± 6.2	16 ± 6	0.5
Anesthesia time (minutes)	24 ± 5.8	23.8 ± 6.3	21 ± 6.2	0.2

(): interquartile range, ¥ test of differences between treatment groups; ANOVA or Kruskal-Wallis for continuous variables, Chi-square for categorical variables. Group G1: fentanyl, group G2: ketamine, group G3: fentanyl + ketamine

**Post operative analgesia:** the median of pain scores on PACU arrival was 2 [IQR=-3 - 8], 1[IQR=-2 - 4], and 1[IQR=-1 - 3] for G1, G2, and G3 groups respectively with no significant difference (Table 2). Further evolution of the pain score showed the superiority of fentanyl-ketamine association at 0, 30 and 60 minutes of recovery period with only a significant difference at 30 min (median FLACC scale G1=3 [IQR=-1 - 7]; median FLACC scale G2=1 [IQR=-2- 4]; median FLACC scale G3=0 [IQR=-1 - 1]; p = 0.009) compared to the other groups (Table 2). At 30 minutes; the LSD test showed that the FLACC pain score was significantly lower in the G3 group (median FLACC scale G3=0 [IQR=-1 - 1]) compared to group G1 (median FLACC scale G1=3 [IQR=-1 - 7]) with p=0.008 and group G2 (median FLACC scale G2=1 [IQR=-2 - 4]) with p=0.03 (Table 2). The request for additional analgesia was greater in the fentanyl group (G1) on arrival to the PACU and 30 minutes after arrival without statistical significance (p=0.1).

**Table 2:** FLACC scale at different times in the PACU

	Group G1	Group G2	Group G3	P-value
0 minutes	2 (-3 - 7)	1 (-2 - 4)	1 (-1 - 3)	0.4
30 minutes	3 (-1 - 7)	1 (-2 - 4)	0 (-1 - 1)	0.09
60 minutes	0 (-1 - 1)	0 (0)	0 (0)	0.1
90 minutes	0 (0)	0 (0)	0 (0)	0.3

(): interquartile range, ¥ Test of differences between treatment groups; Kruskal-Wallis for continuous variables, Chi-square for categorical variables. group G1: fentanyl, group G2: ketamine, group G3: fentanyl + ketamine; FLACC: face, legs, activity, cry, controllability: PACU: post-anesthetic care unit

**Side effects:** three patients had postoperative vomiting: one patient belonged to the group fentanyl (G1), and two others belonged to the group receiving the fentanyl-ketamine association (G3) without statistical differences between the groups (p=0.3). However, ketamine's side effects (nystagmus, hallucinations, agitation) were not observed in our study.

## Discussion

In our study, evaluation of postoperative analgesia in children undergoing tonsillectomy showed that a low dose of ketamine associated with a low dose of fentanyl provides better analgesia in the early postoperative period without causing additional side effects due to higher doses. Tonsillectomy is a commonly performed procedure that is associated with postoperative pain due to the combination of nervous irritation, inflammation, and spasms of the pharyngeal muscles. Thus, more molecules are used for analgesia [8,9]. Anti-inflammatory drugs represent an alternative for analgesia but their effect remains unproven and their use is still limited due to their bleeding risk [5]. Corticosteroids have been used in several studies to reduce pain and PONV by limiting the local inflammatory response and helping early feeding [6,10,11]. The aim of using ketamine as an adjuvant to fentanyl is to reduce opioid prescription and to prevent the adverse effects of opioids while providing adequate analgesia [2,4]. Ketamine for postoperative analgesia is well-acknowledged for both abdominal and thoracic surgery [10,12]. Ketamine is a noncompetitive antagonist on the N-methyl-D-aspartate (NMDA) receptors, which are the cause of postoperative pain and have analgesic properties at sub-anesthetic doses [4,13]. Some authors describe a decrease in pain scores and a significant reduction in morphine consumption following a single low-dose ketamine (0.1 to 0.5 mg/kg) intravenously or intramuscularly [4,14].

In a meta-analysis including 37 studies (2385 patients), Subramaniam *et al*. [15], proved the efficacy of low doses of ketamine used in single injection (0.15 to 1 mg/kg) or continuous perfusion (0.125 to 0, 25 mg/kg per hour), especially in the case of intense and prolonged pain that can lead to tolerance to opioids. A Swedish study showed that the addition of low doses of ketamine to morphine before the hospitalization of patients presenting peripheral fractures provides satisfactory and adequate pain relief [16]. A postoperative low-dose of ketamine for the first 48 hours after abdominal surgery (2 microg x kg (-1) x min (-1)) after a 0.5 mg/kg bolus) was found to improve postoperative analgesia with a significant decrease of morphine consumption, with a lower incidence of nausea, and no side effects of ketamine [17]. In an animal model, Van *et al*. [18] found that pre-incisional treatment with an NMDA antagonist was not more beneficial than post-incisional treatment for postoperative pain relief. The authors also concluded that the analgesic effect of ketamine is better with increasing duration or total dose administration [18]. Our results highlight the benefits of a single injection of ketamine in a short surgical procedure. However, other teams did not find importance in terms of analgesia and morphine consumption with ketamine bolus followed by continuous infusion [6,19]. A randomized-controlled study evaluated the effects of intranasal ketamine (1.5 mg/kg ketamine) and intranasal fentanyl (1.5 mg/kg) for postoperative pain relief after tonsillectomy in children [20].

The authors found that intranasal ketamine and intranasal fentanyl provided significantly stronger analgesic effects compared to intravenous paracetamol administration at postoperative 15, 30, and 60 min in CHEOPS (p < 0.05). The adverse effects of ketamine that limit its use are generally not observed for the lower doses used in analgesia [15,21,22]. None of our patients had delirium or nystagmus. Postoperative nausea and vomiting following adenotonsillectomy may be due to swallowed blood, pain, opioid administration, and direct oropharyngeal irritation [23]. Although ketamine is associated with a significant incidence of vomiting [24], we did not find any significant difference in the incidence of PONV between the three groups. Our results are in agreement with the results of Taheri [11] who compared the adverse effects of ketamine (0.5 mg/kg), and fentanyl (1 μg/kg) in the context of the child's tonsillectomy and found no difference between the two groups regarding the occurrence of PONV. In a published meta-analysis in 2014 by Cho *et al*. it was established that the local or systemic preoperative administration of ketamine could reduce pain without side-effects in children undergoing tonsillectomy and it has also been stated that further clinical trials with clear methodologies are needed [24]. Our study had several limitations. There were many surgeons to operate on children in our study, and we were not able to attribute all tonsillectomies to one surgeon during the study duration, in our university hospital, however, the technique of the surgery was standardized. Despite the use of validated scales (FLAC) to evaluate pain in this age group, sometimes symptoms of pain were not very clear (activity and controllability) mainly in young children because of anxiety in a surgical context.

## Conclusion

Ketamine associated with fentanyl improved more pain-controlling effect than fentanyl or ketamine alone in children undergoing adenotonsillectomy.

### What is known about this topic

- Tonsillectomy is a painful surgery, which is often an ordeal for both children and their parents;
- Ketamine is a potent noncompetitive N methyl-D-aspartate (NMDA) antagonist that when added to general anesthesia at sub-anesthetic doses may reduce the postoperative analgesics consumption;
- Opioids cause drowsiness, respiratory depression, and vomiting in the postoperative period.

### What this study adds

- Ketamine alone had the same effect as fentanyl on postoperative analgesia after tonsillectomy in children;
- Combination of less dose of ketamine and fentanyl provided the most reduction of postoperative pain after tonsillectomy in children;
- Fentanyl or ketamine alone had a less pain-controlling effect in children undergoing adenotonsillectomy.
